# Automated risk assessment of newly detected atrial fibrillation poststroke from electronic health record data using machine learning and natural language processing

**DOI:** 10.3389/fcvm.2022.941237

**Published:** 2022-07-29

**Authors:** Sheng-Feng Sung, Kuan-Lin Sung, Ru-Chiou Pan, Pei-Ju Lee, Ya-Han Hu

**Affiliations:** ^1^Division of Neurology, Department of Internal Medicine, Ditmanson Medical Foundation Chiayi Christian Hospital, Chiayi City, Taiwan; ^2^Department of Nursing, Min-Hwei Junior College of Health Care Management, Tainan, Taiwan; ^3^School of Medicine, National Taiwan University, Taipei, Taiwan; ^4^Clinical Data Center, Department of Medical Research, Ditmanson Medical Foundation Chiayi Christian Hospital, Chiayi City, Taiwan; ^5^Department of Information Management and Institute of Healthcare Information Management, National Chung Cheng University, Chiayi County, Taiwan; ^6^Department of Information Management, National Central University, Taoyuan, Taiwan

**Keywords:** atrial fibrillation, electronic health records, ischemic stroke, natural language processing, prediction

## Abstract

**Background:**

Timely detection of atrial fibrillation (AF) after stroke is highly clinically relevant, aiding decisions on the optimal strategies for secondary prevention of stroke. In the context of limited medical resources, it is crucial to set the right priorities of extended heart rhythm monitoring by stratifying patients into different risk groups likely to have newly detected AF (NDAF). This study aimed to develop an electronic health record (EHR)-based machine learning model to assess the risk of NDAF in an early stage after stroke.

**Methods:**

Linked data between a hospital stroke registry and a deidentified research-based database including EHRs and administrative claims data was used. Demographic features, physiological measurements, routine laboratory results, and clinical free text were extracted from EHRs. The extreme gradient boosting algorithm was used to build the prediction model. The prediction performance was evaluated by the C-index and was compared to that of the AS5F and CHASE-LESS scores.

**Results:**

The study population consisted of a training set of 4,064 and a temporal test set of 1,492 patients. During a median follow-up of 10.2 months, the incidence rate of NDAF was 87.0 per 1,000 person-year in the test set. On the test set, the model based on both structured and unstructured data achieved a C-index of 0.840, which was significantly higher than those of the AS5F (0.779, *p* = 0.023) and CHASE-LESS (0.768, *p* = 0.005) scores.

**Conclusions:**

It is feasible to build a machine learning model to assess the risk of NDAF based on EHR data available at the time of hospital admission. Inclusion of information derived from clinical free text can significantly improve the model performance and may outperform risk scores developed using traditional statistical methods. Further studies are needed to assess the clinical usefulness of the prediction model.

## Introduction

Ischemic stroke is associated with a substantial risk of recurrence with a one-year recurrence rate ranging from 6 to 18% (1–4). The risk of stroke recurrence depends on the subtypes of ischemic stroke. As compared to other stroke subtypes, the recurrence rate of cardioembolic stroke is relatively high (5, 6). Moreover, cardioembolic strokes are often followed by strokes of the same type (6, 7). Atrial fibrillation (AF) is the most common cause of cardioembolic stroke, and even embolic stroke of undetermined source (ESUS) may originate from subclinical AF (8). As the population ages, AF-related strokes have increased and may triple in the next few decades (9, 10). Fortunately, the advancement of non-vitamin K antagonist oral anticoagulant therapy has made great progress in preventing patients with AF from cardioembolic stroke (8). Nonetheless, since AF can be paroxysmal, it may go undetected and therefore undiagnosed in patients undergoing routine electrocardiography (ECG) examinations. In fact, for ischemic stroke patients with undiagnosed AF, delayed use of oral anticoagulants may double the risk of recurrent stroke or transient ischemic attack (TIA) (11). Considering the impact of anticoagulant therapy on the outcome, poststroke screening for AF is thus critical for preventing recurrent stroke in patients with acute ischemic stroke (AIS).

Approximately 30% of all ischemic strokes are without any apparent cause (12). Among these cryptogenic strokes, nearly two-thirds are considered to stem from embolism (12). A study points out that through a series of heart rhythm monitoring, AF can be detected in up to 24% of patients with AIS or TIA (13). In addition to 24-h or even 72-h Holter monitoring (14), numerous studies have established that extended ECG monitoring *via* either implantable or external devices increases the yield of AF detection in patients with AIS (15, 16). However, given the limited medical resources, setting the right priorities of extended ECG monitoring by stratifying patients into different risk groups likely to have newly detected AF (NDAF) is more crucial than implementing population-level screening (17).

To date, more than twenty risk scores have been proposed to assess the risk of poststroke NDAF (18, 19). These risk scores vary in their complexity, target population, outcome definition, predictor variables, and ease of implementation. Most risk scores were derived or validated in patients with AIS while some of them were derived from a specific population with cryptogenic stroke or ESUS (20, 21). The simplest risk score consists of only two predictor variables, that is, age and stroke severity as assessed using the National Institutes of Health Stroke Scale (NIHSS) (22). Nevertheless, many of the risk scores require additional diagnostic work-up or interpretation of examination results to obtain the necessary predictors, such as markers of blood, ECG, echocardiography, as well as brain and vascular imaging (18). Routine use of such risk scores may be impractical in the context of the extra time and cost required.

On the other hand, with the ubiquitous use of electronic health records (EHRs) and the advancement in computational power, it has become feasible to use EHRs for the creation, validation, and implementation of data-driven risk prediction models (23, 24). For example, a previous study developed and validated an EHR-based prediction tool for 5-year AF risk in the general population (25), demonstrating a simple and cost-conscious approach to AF screening. Furthermore, in addition to structured numerical and categorical data, EHRs accommodate a multitude of unstructured textual data such as narrative clinical notes. Combining information extracted from clinical free text through natural language processing with structured data has shown promising results in improving the performance of risk prediction models (26–28).

AF-related strokes tend to be more severe and may manifest with different clinical features than other subtypes of ischemic strokes (29, 30). A higher risk of NDAF has been observed in patients with greater stroke severity (22, 31). Previous studies have shown that information extracted from clinical text can be used to represent patients' stroke severity (28, 32). Furthermore, stroke patients with AF have a higher prevalence of heart diseases and experience more cardiac events than those without AF (29–31). Symptoms, signs, or examinations related to heart diseases are typically documented in clinical notes. However, such information may not be captured or routinely collected as structured data in the EHR system. We thereby hypothesized that clinical text contains information that can discriminate between strokes stemming from AF and those not stemming from AF. In this study, we aimed to develop an EHR-based machine learning (ML) model to assess the risk of NDAF. To this end, we investigated various ML models using structured data, unstructured textual data, or a combination of both. In addition, the prediction performance of the developed ML models was compared to that of two traditional risk scores on a temporal test set of patients hospitalized for AIS.

## Materials and Methods

### Data sources

The study data was obtained from the stroke registry of the Ditmanson Medical Foundation Chia-Yi Christian Hospital and the Ditmanson Research Database (DRD), a deidentified database comprising both EHR data and administrative claims data for research purposes. The DRD currently holds clinical information of over 1.4 million patients. The hospital stroke registry has prospectively enrolled consecutive hospitalized stroke patients since 2007 conforming to the design of the nationwide Taiwan Stroke Registry (33). To create the dataset for this study, we linked the stroke registry to the DRD using a unique encrypted patient identifier. Information regarding risk factors and stroke severity as assessed using the NIHSS was obtained from the stroke registry. Billing information and medical records from 2 years before to 1 year after the index stroke were extracted from the DRD.

The study protocol was approved by the Ditmanson Medical Foundation Chia-Yi Christian Hospital Institutional Review Board (IRB2020135). The requirement for informed consent was waived because of the retrospective design. The study protocol conforms to the ethical guidelines of the 1975 Declaration of Helsinki.

### Study population

The study population selection is shown in Supplementary Figure 1. The stroke registry was queried for all hospitalizations for AIS between Oct 2007 and Sep 2020. Only the first hospitalization was included for each patient. Patients who suffered an in-hospital stroke or whose records could not be linked were excluded. The study population was split into a training set (patients admitted before the end of 2016) and a temporal test set (those admitted from 2017 onwards). All patients were traced in the DRD until AF was detected, death, the last visit within 1 year after the index stroke, or February 28, 2021, whichever came first.

### Predictor and outcome variables

**Figure 1:**
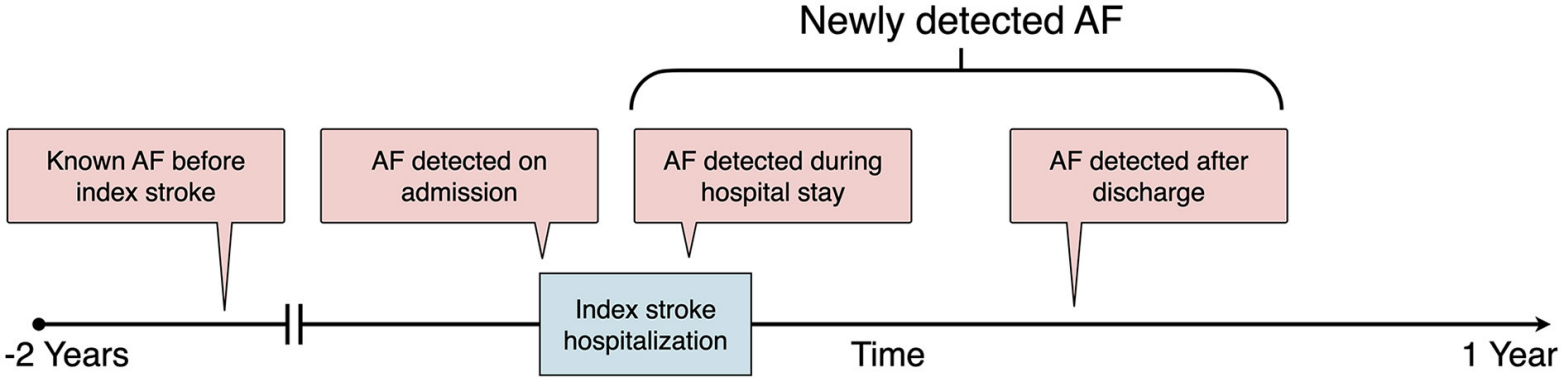
Definition of AF categories according to the time sequence between AF detection and the index stroke. AF, atrial fibrillation.

The class label (outcome) was AF, which was defined according to an AF ascertainment algorithm detailed in the Supplementary Methods in the Supplementary Material. According to the time sequence between AF detection and the index stroke (13), AF was further categorized as known AF before the index stroke, AF detected on admission, AF detected during the index stroke hospitalization, and AF detected after discharge (Figure 1). During the training phase, we trained ML models to predict which stroke is likely to stem from AF. Therefore, patients with all kinds of AF were retained in the training set. Because the study purpose was to build an ML model to assess the risk of NDAF poststroke, i.e., AF detected during the index stroke hospitalization and AF detected after discharge (Figure 1), patients who had known AF before the index stroke or AF detected on admission (34) were further excluded from the test set.

A total of 20 structured predictor variables (Supplementary Table 3), including age, sex, body mass index (BMI), vital signs, and results of routine blood tests, were chosen because they are readily available from EHRs upon admission. Missing values were imputed as mean values for continuous variables. Besides these structured variables, the free text extracted from the History of Present Illness section of the admission note was preprocessed through the following steps: spell checking, abbreviation expansion, removal of non-word symbols, removal of words suggestive of AF (“paroxysmal”, “atrial”, “fibrillation”), lowercase conversion, lemmatization, marking of negated words with the suffix “_NEG” using the Natural Language Toolkit mark_negation function, and stop-word removal.

**Figure 2:**
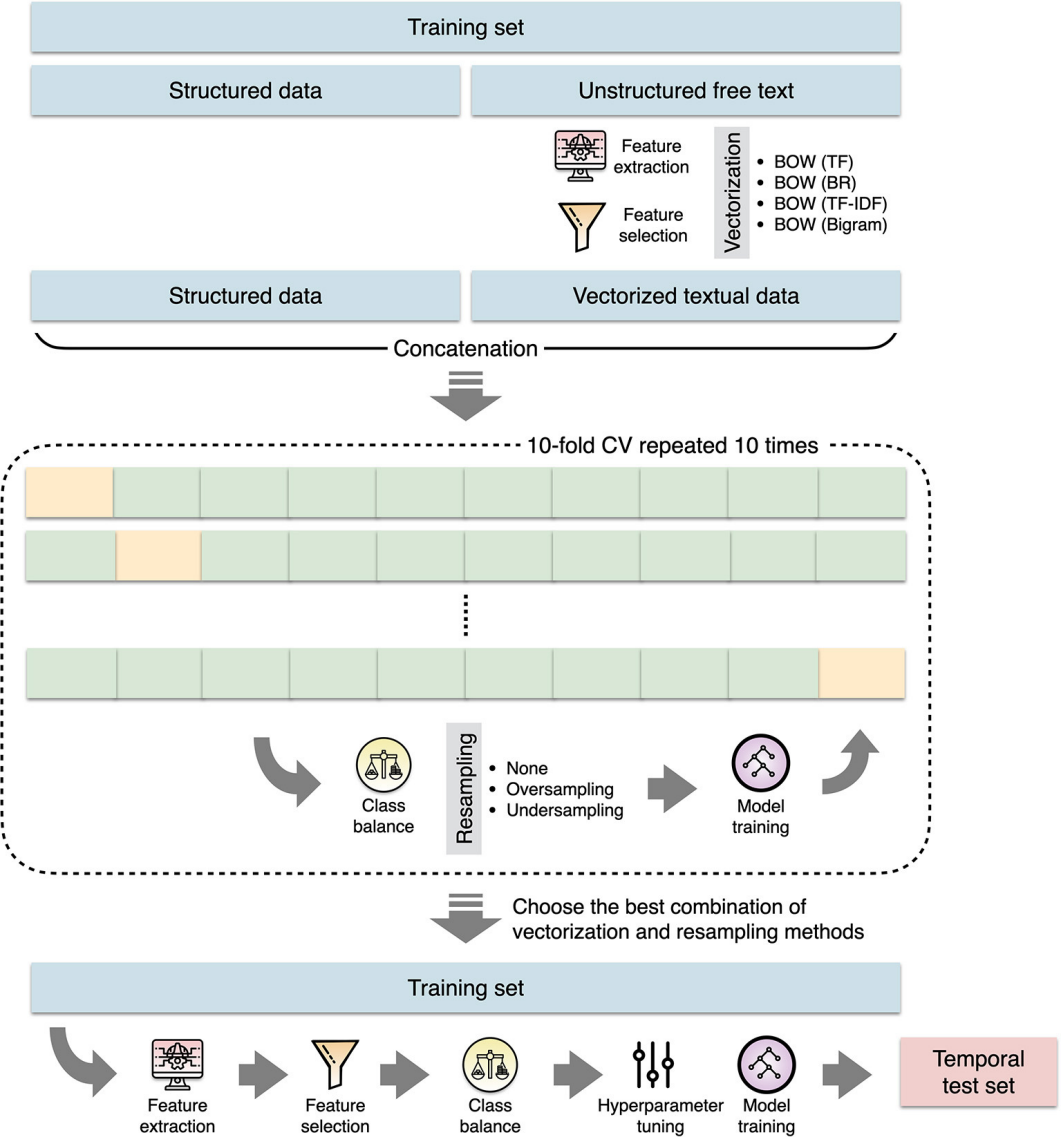
The process of machine learning model construction. BOW, bag-of-words; BR, binary representation; CV, cross validation; TF, term frequency; TF-IDF, term frequency with inverse document frequency.

The preprocessed text was then vectorized using the bag-of-words (BOW) approach with three different types of feature representation (Figure 2). We built a document-term matrix in which each column represents each unique feature (word) from the text corpus while the rows represent each document (present illness for each patient). The cells represent the counts of each word within each document (term frequency), the absence or presence of each word within each document (binary representation), or the term frequency with inverse document frequency (TF-IDF) weighting (35). Because medical terms are commonly comprised of two words or even more, we further experimented with adding word bigram features (two-word phrases) to the basic BOW model. To reduce noises such as redundant and less informative features as well as to improve training efficiency (36), we performed feature selection by filtering out words that appeared in <5% of all documents in the training set, followed by performing a penalized logistic regression with 10-fold cross-validation to identify the most predictive words (37).

### Baseline models

For comparison with ML models, we only considered traditional risk scores that are based on variables available from EHRs upon admission. According to a validation study that evaluated eight such risk scores, two risk scores performed better than the others, demonstrating adequate discrimination and calibration (19). These two risk scores were thus used as the baseline models. The AS5F score, composed by age and NIHSS, was developed and validated in cohorts of patients who underwent extended Holter monitoring after AIS or TIA (22). The CHASE-LESS score was constructed from patients hospitalized for AIS in a claims database (31). It comprises seven components, including age, NIHSS, as well as the presence of coronary artery disease, congestive heart failure, hyperlipidemia, diabetes, and prior stroke or TIA.

### Machine learning models

ML models were constructed by using structured data, vectorized textural data, or a combination of both (Figure 2). Because class imbalance might influence the classification performance, we experimented with resampling methods to maintain the ratio of majority and minority classes as 1:1, 2:1, or 3:1 (38). The extreme gradient boosting (XGB) algorithm was used to build classifiers. The XGB classifier trains a series of classification and regression trees where each successive tree attempts to correct the errors of the preceding trees.

During the training process, we first evaluated a suite of different combinations of text vectorization techniques and resampling methods without hyperparameter tuning. We repeated 10-fold cross-validation 10 times to obtain the performance estimates. The area under the receiver operating characteristic curve (AUC) was used as the evaluation metric because both positive and negative classes are important. After the optimal combination of text vectorization and resampling methods was determined, ML models were trained from the full training set through feature extraction, feature selection, class balancing, followed by hyperparameter tuning. Hyperparameter optimization for each model was performed by repeating 10-fold cross-validation 10 times. Model error was minimized in terms of AUC. We performed a grid search to find optimal hyperparameters following steps proposed in a prior study (39). After building the XGB classifiers, we used Shapley additive explanations (40) to interpret the output of the XGB classifiers. The experiments were carried out by using scikit-learn, XGBoost, imbalanced-learn, and SHAP libraries within Python 3.7 environment.

### Statistical analysis

Categorical variables were reported with counts and percentages. Continuous variables were presented as means with standard deviations or medians and interquartile ranges. Differences between groups were tested by Chi-square tests for categorical variables and *t* tests or Mann-Whitney U tests for continuous variables, as appropriate.

The incidence rate of NDAF was expressed as events per 1,000 person-years. To assess the prediction performance of each prediction model, Cox proportional hazard regression analyses were performed by entering each risk score or the predicted probability output by each ML model as a continuous variable. Harrell's concordance index (C-index) was calculated to evaluate and compare model performance. The C-index ranges from 0.5 to 1.0, with 0.5 indicating random guess and 1 indicating perfect model discrimination. A model with a C-index value above 0.7 is considered acceptable for clinical use (41).

All statistical analyses were performed using Stata 15.1 (StataCorp, College Station, Texas) and R version 4.1.1 (R Foundation for Statistical Computing, Vienna, Austria). Two-tailed *p* values were considered statistically significant at < 0.05.

## Results

### Characteristics of the study population

A total of 6,321 patients were eligible for this study (Supplementary Figure 1). The training set consisted of 4,604 patients who were admitted before the end of 2016. Among patients in the training set, 422 (9.2%) had known AF, 265 (5.6%) were diagnosed with AF on admission, and 232 (5.0%) developed NDAF during follow-up. Among 1,717 patients who were admitted from 2017 onwards, 122 and 103 were excluded because of having known AF before the index stroke and being diagnosed with AF on admission, respectively. Therefore, the temporal test set consisted of 1,492 patients. During a median follow-up of 10.2 months, 87 (5.8%) patients in the temporal test set were identified as having NDAF. Each patient had an average of 3.1 hospital visits per month during the follow-up period. The incidence rate of NDAF was 87.0 per 1,000 person-year. Table 1 lists the characteristics of the patients. Patients in the training set were older, more likely to be female, less likely to have diabetes mellitus, and tended to have hypertension, coronary artery disease, congestive heart failure, as well as prior stroke or TIA. They also had significantly higher NIHSS, AS5F, and CHASE-LESS scores.

**Table 1:** Characteristics of the study population.

**Characteristic**	**Training set** **(*****N*** = **4,604)**	**Temporal test set** **(*****N*** = **1,492)**	***P***
Age, mean (SD)	69.2 (12.3)	68.0 (13.5)	=0.002
Female	1,896 (41.2)	531 (35.6)	= <0.001
Hypertension	3,705 (80.5)	1,119 (75.0)	= <0.001
Diabetes mellitus	1,958 (42.5)	683 (45.8)	=0.028
Hyperlipidemia	2,670 (58.0)	852 (57.1)	=0.546
Coronary artery disease	560 (12.2)	103 (6.9)	= <0.001
Congestive heart failure	228 (5.0)	25 (1.7)	= <0.001
Prior stroke or TIA	1,143 (24.8)	274 (18.4)	= <0.001
NIHSS, median (IQR)	5 (3-10)	5 (2-8)	= <0.001
AS5F, median (IQR)	67.4 (59.2–76.5)	65.8 (56.9–74.2)	= <0.001
CHASE-LESS, median (IQR)	6 (5-8)	6 (4-7)	= <0.001

*Data are numbers (percentage) unless specified otherwise*.

*IQR, interquartile range; NIHSS, National Institutes of Health Stroke Scale; SD, standard deviation; TIA, transient ischemic attack*.

### Performance of prediction models

**Figure 3:**
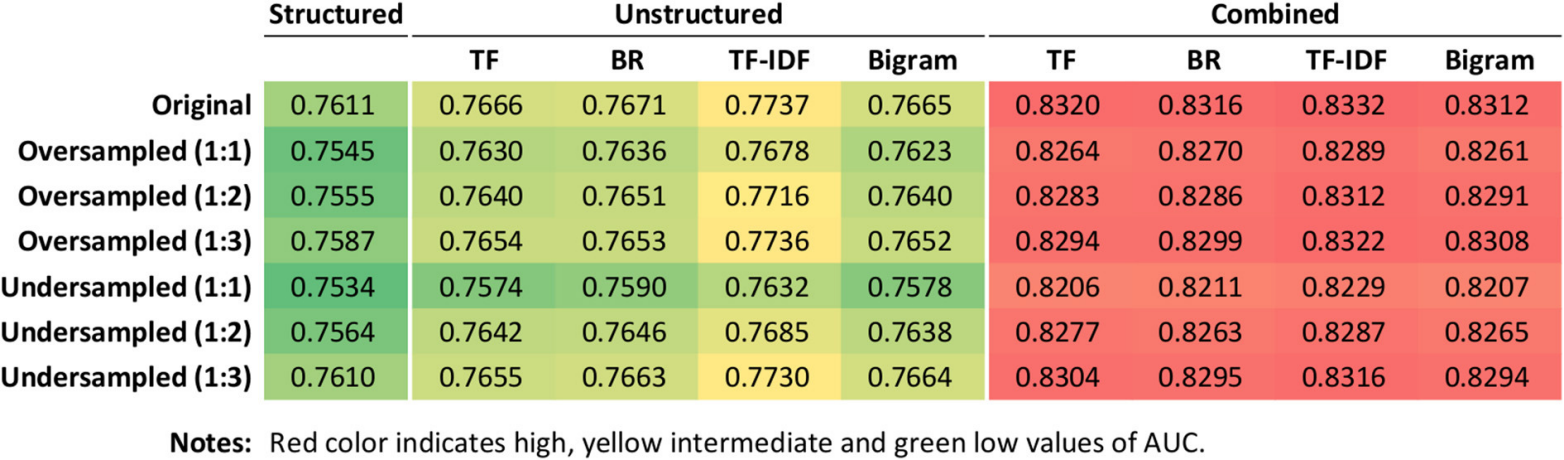
Heat map showing AUC values across machine learning models with different combinations of text vectorization techniques and resampling methods. AUC, area under the receiver operating characteristic curve; BR, binary representation; TF, term frequency; TF-IDF, term frequency with inverse document frequency.

According to the estimates of AUC obtained from the 10 times of 10-fold cross-validation (Figure 3), ML models using a combination of both structured and unstructured data achieved higher AUCs than those using structured or unstructured data alone. Data resampling did not improve the performance of models. Text vectorization using BOW with TF-IDF weighting generally performed higher than the other feature value representation methods. Therefore, we used the full original training set to build three ML models, that is, a model based on structured data (model A), a model based on textual data vectorized using BOW with TF-IDF weighting (model B), and a model based on both structured data and unstructured textual data vectorized using BOW with TF-IDF weighting (model C).

Table 2 lists the performance of prediction models. All the prediction models significantly predicted the risk of NDAF. Among the ML models, model C had the highest C-index (0.840), which was significantly higher than those of model A (0.791, *p* = 0.009) and model B (0.738, *p* < 0.001). Model C outperformed the AS5F (0.779, *p* = 0.023) and CHASE-LESS (0.768, *p* = 0.005) scores. The C-index of model A was comparable to those of AS5F (*p* = 0.715) and CHASE-LESS (*p* = 0.487) scores. Although model B attained the lowest C-index, its performance was also comparable to the AS5F (*p* = 0.163) and CHASE-LESS (*p* = 0.282) scores.

**Table 2:** Performance of prediction models for predicting newly detected atrial fibrillation.

**Risk score**	**HR (95% CI)**	***P***	**Schoenfeld's global test**	**C-index (95% CI)**
AS5F	=1.10 (1.08–1.13)	= <0.001	=0.062	=0.779 (0.734–0.825)
CHASE-LESS	=1.49 (1.38–1.60)	= <0.001	=0.296	=0.768 (0.721–0.816)
Model A (structured)	=1.05 (1.04–1.06)	= <0.001	=0.764	=0.791 (0.745–0.836)
Model B (unstructured)	=1.04 (1.03–1.05)	= <0.001	=0.060	=0.738 (0.688–0.788)
Model C (combined)	=1.05 (1.04–1.06)	= <0.001	=0.600	=0.840 (0.803–0.876)

*CI, confidence interval; HR, hazard ratio*.

### Model interpretation

**Figure 4:**
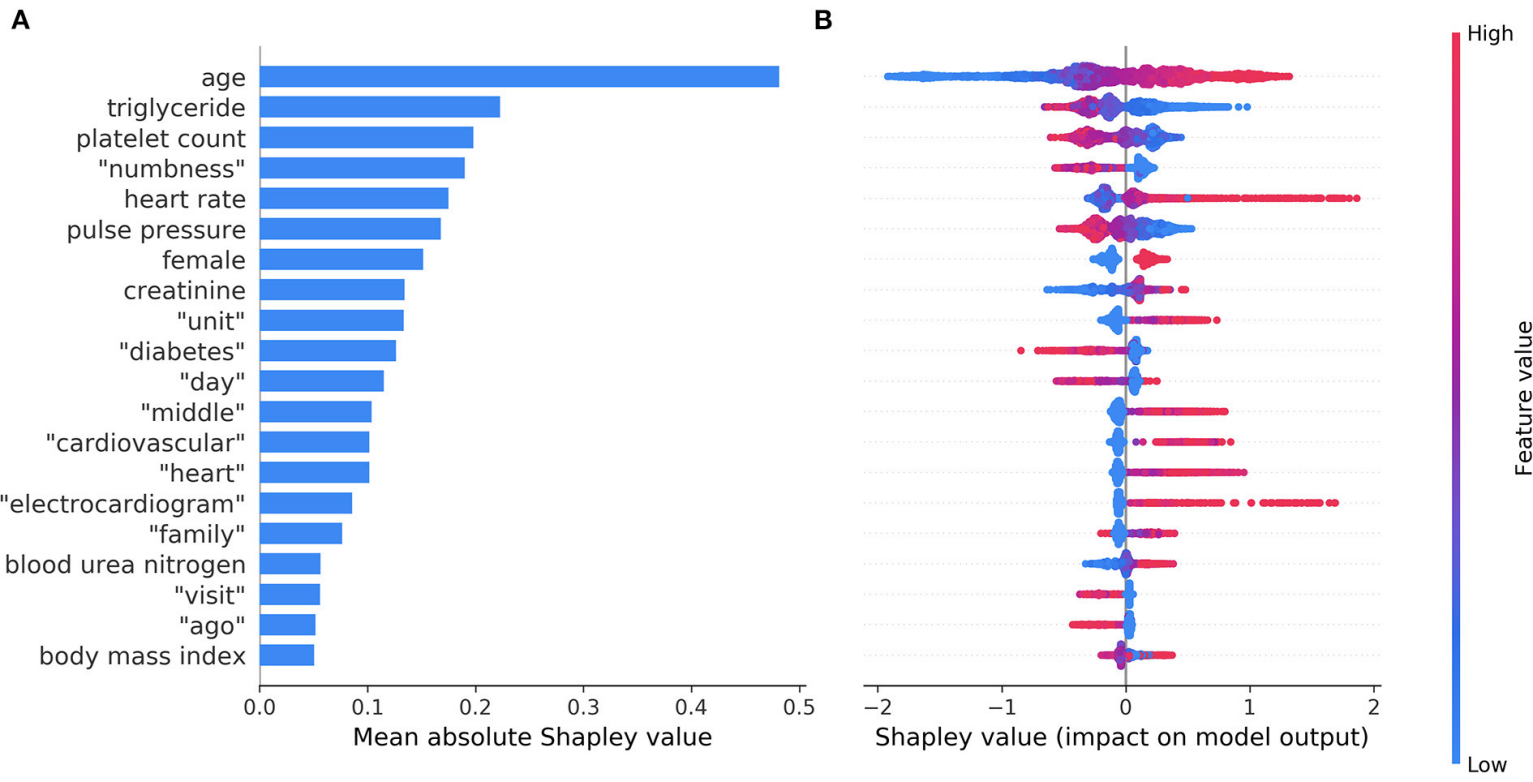
The top 20 most important features identified by the model based on both structured data and unstructured textual data. The mean absolute Shapley values that indicate the average impact on model output are shown in a bar chart **(A)**. The individual Shapley values for these features for each patient are depicted in a beeswarm plot **(B)**, where a dot's position on the x-axis denotes each feature's contribution to the model prediction for that patient. The color of the dot specifies the relative value of the corresponding feature.

Figure 4A shows the top 20 most important features in model C ordered by the mean absolute Shapley value, which indicates the global importance of each feature on the model output. Figure 4B presents the beeswarm plot depicting the Shapley value for every patient across these features, demonstrating each feature's contribution to the model output. According to the magnitude and direction of the Shapley value, patients who were female and those with increased age, high heart rate, elevated creatinine, elevated blood urea nitrogen, and high BMI were more likely to have NDAF. Patients with high triglyceride, platelet count, and pulse pressure were less likely to have NDAF. Words associated with an increased risk of NDAF included “unit”, “middle”, “cardiovascular”, “heart”, “electrocardiogram”, and “family”, whereas those associated with a decreased risk were “numbness”, “diabetes”, “day”, “visit”, and “ago”.

The top 20 most important features in model A and model B are shown in Supplementary Figures 2, 3, respectively. The important structured and unstructured predictors identified in model C were generally consistent with those identified separately in model A (structured data) and model B (unstructured textual data).

## Discussion

We found that prediction of NDAF using routinely collected variables from EHRs was feasible. ML models performed better than or were comparable to existing risk scores. The ML model based on both structured variables and text had higher discriminability than those of AS5F and CHASE-LESS scores. Furthermore, by using the Shapley value to reveal the significance of features, we identified important predictors of NDAF that may help gain insight into clinical practice for stroke prevention.

### Important predictors of newly detected atrial fibrillation

Many studies have investigated prediction models for NDAF in the general population (25, 42, 43) or in selected patient groups such as those with stroke or TIA (18, 21, 22, 31, 44, 45). Owing to the different characteristics of at-risk populations, it is arguable whether the relationships between the predictors and NDAF are similar across patient groups. Among the identified structured predictor variables, some of them such as age and BMI were common to the general population and patients with stroke (18, 42), others are known predictors in the general population but have seldom been used to predict poststroke NDAF, while still others are controversial predictors that warrant further study. For example, chronic kidney disease is a positive predictor whereas hyperlipidemia is a negative predictor of NDAF in the general population (25, 43). This study echoes those findings by showing positive associations of NDAF with elevated creatinine, elevated blood urea nitrogen, as well as decreased triglyceride level (Figure 4B). On the other hand, the evidence on the relationship between heart rate and NDAF is conflicting (46).

The central hypothesis of this study is that clinical free text contains information that may be used to predict NDAF. We indeed identified several words that could help make predictions. The reason why some of these words were associated with the risk of NDAF may be obscure at first glance but could be revealed by examining each word in its context. For example, the word “unit” from the term “intensive care unit” and the word “middle” from the term “middle cerebral artery infarction” typically imply severe stroke, which is a known predictor of NDAF (22, 31). These results demonstrate that useful and informative predictors could be derived from unstructured text in EHRs without intervening human curation. Despite this, since clinicians may use different terms to describe the same condition in clinical text, the relationship between such terms might not be accurately represented. Concept-based feature extraction using specialized medical ontologies can be explored in future research (35).

### Advantages of EHR-based machine learning models

Traditional prediction models used in clinical practice are generally built on limited predefined variables using logistic regression. Although such models have reasonable prediction performance, whether they are applicable in routine clinical practice and relevant to a specific context is yet to be determined (47). First, logistic regression models necessitate the assumptions of linear and additive relationships among predictors being fulfilled, while ML algorithms, especially tree-based models, are more effective in capturing potential nonlinear relationships and handling complex interactions between the predictor and outcome variables (48). Second, considering the wide variety of data in EHRs, data-driven prediction modeling may allow identifying novel predictors in the context of insufficient prior knowledge of the real system (49). In this respect, ML is suitable for building complex models and analyzing noisy data such as that stored in EHRs (50). ML techniques were also applied to predict cardioembolic vs. non-cardioembolic stroke mechanism in patients with ESUS (51). Recently, deep learning techniques have been introduced to predict new-onset AF in the general population using structured primary care data or unstructured 12-lead ECG traces (52, 53).

### Clinical applications and significance

Poststroke AF screening is essential for choosing the optimal strategy for secondary stroke prevention. However, to be resource efficient, extended ECG monitoring should be prioritized for patients at a high risk of NDAF. The developed ML model will be suited for assessing the risk of individual patients and assisting in personalized clinical decisions. Moreover, locally constructed prediction models may be more suitable for real-world clinical use than externally developed risk models (25). Since the prediction model was derived from EHRs, it is ideal to implement this model in the EHR as a decision support tool. With this tool, the calculation of risk estimates and the flagging of high-risk patients can be automated within the EHR, streamlining the process of risk stratification for poststroke AF screening.

### Limitations

This study has several limitations. First, patients were traced through EHRs. Because patients might be diagnosed with AF outside the study hospital, some outcome misclassification was inevitable. Nevertheless, the frequent visits to the study hospital observed in this stroke population (>3 visits per month) might have alleviated this problem. Second, the diagnosis of AF was made in usual-care settings, where AF was detected almost exclusively by 12-lead ECG or 24-h Holter ECG. Advanced ECG monitoring *via* either implantable or external devices to detect subclinical or low-burden AF was not used. Consequently, the study findings are valid for relatively high-burden AF (54). Third, although data-driven ML modeling has its own advantages, the predictor-outcome relationships discovered from data does not mean causality. In other words, prediction accuracy should not be equated to causal validity (55). Fourth, as this is a single-site study, the generalizability of the study findings may be restricted. Variations in the terminology used in clinical documentation are to be expected across healthcare settings. However, the methods used here may allow other healthcare systems to develop their own customized versions of prediction models.

### Conclusions

It is feasible to build an ML model to predict NDAF based on EHR data available at the time of hospital admission. Inclusion of information derived from clinical free text can significantly improve the model performance and may outperform risk scores developed using traditional statistical methods. These improvements may be due to both the modeling approach to delineate nonlinear decision boundaries and the use of textual features that help characterize nuances of disease presentation across patients. Despite these findings, further studies are required to confirm the approach's generalizability and the clinical usefulness of the prediction model.

## Data Availability Statement

The data used in this study cannot be made available because of restrictions regarding the use of EMR data. Requests to access these datasets should be directed to Y-HH, yhhu@mgt.ncu.edu.tw.

## Ethics Statement

The studies involving human participants were reviewed and approved by Ditmanson Medical Foundation Chia-Yi Christian Hospital Institutional Review Board. Written informed consent for participation was not required for this study in accordance with the national legislation and the institutional requirements.

## Author contributions

Study concept and design: S-FS and Y-HH. Acquisition of data: S-FS and R-CP. Drafting of the manuscript: S-FS and K-LS. Study supervision: P-JL and Y-HH. Analysis and interpretation of data and critical revision of the manuscript for important intellectual content: all authors. All authors had full access to all the data in the study and take responsibility for the integrity of the data and the accuracy of the data analysis.

## Funding

This research was supported in part by the Ditmanson Medical Foundation Chia-Yi Christian Hospital-National Chung Cheng University Joint Research Program (Grant number CYCH-CCU-2022-03).

## Conflict of interest

The authors declare that the research was conducted in the absence of any commercial or financial relationships that could be construed as a potential conflict of interest.

## Publisher's note

All claims expressed in this article are solely those of the authors and do not necessarily represent those of their affiliated organizations, or those of the publisher, the editors and the reviewers. Any product that may be evaluated in this article, or claim that may be made by its manufacturer, is not guaranteed or endorsed by the publisher.
