# Supplementary material for: Automated risk assessment of newly detected atrial fibrillation poststroke from electronic health record data using machine learning and natural language processing
Source: Front Cardiovasc Med. 2022 Jul 29;9:941237. doi: 10.3389/fcvm.2022.941237 (PMC9372298; doi:10.3389/fcvm.2022.941237)
Supplement: Supplementary file 1 [file Data_Sheet_1.PDF]

# Supplementary Material

## 1 Supplementary Methods

Based on a previous study (1), we constructed various component algorithms and composite algorithms to ascertain AF status (Supplementary Table 1). These algorithms were used to automate AF ascertainment from the database using both structured variables and unstructured clinical text. Diagnostic codes, medication codes, physicians' notes, electrocardiography (ECG) reports, and echocardiography reports were retrieved from the Ditmanson Research Database. Free text in the notes and reports were screened for mentions of AF-related keywords including “atrial fibrillation”, “AF”, “Afib”, and “PAF”. The NegEx algorithm was used to determine whether the mention was positive or negative (2). By applying these algorithms, the AF status of each patient before discharge from the stroke hospitalization was determined using all available information up until the discharge date. The medical records of a random sample of 1000 patients were manually reviewed independently by two experienced stroke clinicians to adjudicate AF and any discrepancy was resolved by consensus.

The performance of each algorithm was evaluated by estimating the sensitivity, specificity, positive predictive value, negative predictive value, and the area under the receiver operating characteristic curve (AUC). The algorithm that had the highest AUC value was used to ascertain AF status in the whole study population. Supplementary Table 2 shows the performance of various AF ascertainment algorithms. The highest AUC (0.990) was attained by the composite algorithm that defines AF as any positive mention of AF-related keywords in ECG or echocardiography reports, or presence of diagnostic codes of AF (AF-D in Supplementary Tables 1 and 2). This algorithm was thus chosen for ascertaining AF status.

**Supplementary Table 1. Definition of various algorithms to ascertain AF status.**

| Algorithm | Definition |
|---|---|
| Component | |
| AF-1 | Any positive mention of "atrial fibrillation", "AF", "Afib", or "PAF" in ECG reports |
| AF-2 | Any positive mention of "atrial fibrillation", "AF", "Afib", or "PAF" in echocardiography reports |
| AF-3 | Any positive mention of "atrial fibrillation", "AF", "Afib", or "PAF" in physicians' notes |
| AF-4 | Diagnostic codes for AF (ICD-9-CM 427.31 or ICD-10-CM I48.91) |
| AF-5 | On antiarrhythmic medications (amiodarone, dronedarone, or propafenone) |
| AF-6 | On oral anticoagulants (warfarin, apixaban, edoxaban, rivaroxaban, or dabigatran) |
| Composite | |
| AF-A | AF-1 or AF-4 |
| AF-B | AF-1 or AF-3 |
| AF-C | AF-3 or AF-4 |
| AF-D | AF-1 or AF-2 or AF-4 |
| AF-E | AF-1 or AF-3 or AF-4 |
| AF-F | AF-1 or AF-2 or AF-3 or AF-4 |
| AF-G | AF-1 or AF-2 or AF-3 or AF-5 |
| AF-H | AF-1 or AF-2 or AF-3 or AF-6 |
| AF-I | AF-1 or AF-2 or AF-3 or AF-5 or AF-6 |
| AF-J | AF-1 or AF-2 or AF-3 or AF-4 or AF-5 or AF-6 |

AF, atrial fibrillation; ECG, electrocardiography; ICD-9-CM, International Classification of Diseases, Ninth Revision, Clinical Modification; ICD-10-CM, International Classification of Diseases, Tenth Revision, Clinical Modification.

**Supplementary Table 2. Performance of various algorithms to ascertain AF status.**

| Algorithm | Sen | Spe | PPV | NPV | AUC (95% CI) |
|---|---|---|---|---|---|
| Component | | | | | |
| AF-1 | 89.3% | 100.0% | 100.0% | 97.9% | 0.947 (0.923–0.970) |
| AF-2 | 50.3% | 100.0% | 100.0% | 90.8% | 0.751 (0.714–0.789) |
| AF-3 | 96.4% | 97.6% | 89.1% | 99.3% | 0.970 (0.955–0.985) |
| AF-4 | 78.7% | 99.8% | 98.5% | 95.8% | 0.892 (0.861–0.923) |
| AF-5 | 33.1% | 99.2% | 88.9% | 87.9% | 0.661 (0.626–0.697) |
| AF-6 | 42.6% | 98.9% | 88.9% | 89.4% | 0.708 (0.670–0.745) |
| Composite | | | | | |
| AF-A | 95.9% | 99.8% | 98.8% | 99.2% | 0.978 (0.963–0.993) |
| AF-B | 100.0% | 97.6% | 89.4% | 100.0% | 0.988 (0.983–0.993) |
| AF-C | 97.0% | 97.5% | 88.6% | 99.4% | 0.973 (0.959–0.986) |
| AF-D | 98.2% | 99.8% | 98.8% | 99.6% | 0.990 (0.980–1.000) |
| AF-E | 100.0% | 97.5% | 88.9% | 100.0% | 0.987 (0.982–0.993) |
| AF-F | 100.0% | 97.5% | 88.9% | 100.0% | 0.987 (0.982–0.993) |
| AF-G | 100.0% | 96.8% | 86.2% | 100.0% | 0.984 (0.978–0.990) |
| AF-H | 100.0% | 96.5% | 85.4% | 100.0% | 0.983 (0.976–0.989) |
| AF-I | 100.0% | 95.7% | 82.4% | 100.0% | 0.978 (0.971–0.985) |
| AF-J | 100.0% | 95.7% | 82.4% | 100.0% | 0.978 (0.971–0.985) |

AF, atrial fibrillation; AUC, area under the receiver operating characteristic curve; CI, confidence interval; NPV, negative predictive value; PPV, positive predictive value; Sen, sensitivity, Spe, specificity.

**Supplementary Table 3. Structured data used for machine learning model construction in the training set (N = 4604).**

| Variable | Value | Missing, n (%) |
|---|---|---|
| Age | 69.2 (12.3) | 0 (0%) |
| Female, n (%) | 1896 (41.2) | 0 (0%) |
| Body mass index, kg/m$^2$ | 24.6 (4.0) | 0 (0%) |
| Systolic BP, mmHg | 164.0 (30.8) | 0 (0%) |
| Diastolic BP, mmHg | 91.3 (17.7) | 0 (0%) |
| Pulse pressure, mmHg | 72.7 (23.2) | 0 (0%) |
| Body temperature, °C | 36.3 (0.7) | 0 (0%) |
| Heart rate, beats per minute | 81.8 (17.9) | 0 (0%) |
| Respiratory rate, breaths per minute | 19.4 (1.8) | 2 (0.04%) |
| Glucose, mg/dL | 164.3 (83.1) | 7 (0.15%) |
| Blood urea nitrogen, mg/dL | 20.1 (11.1) | 149 (3.24%) |
| Creatinine, mg/dL | 1.25 (1.12) | 34 (0.74%) |
| AST, U/L | 29.6 (32.3) | 1134 (24.6%) |
| ALT, U/L | 28.2 (30.3) | 357 (7.75%) |
| Total cholesterol, mg/dL | 187.3 (44.4) | 100 (2.17%) |
| Triglyceride, mg/dL | 135.7 (106.2) | 93 (2.02%) |
| Hemoglobin, g/dL | 13.7 (2.1) | 1 (0.02%) |
| White blood cells count, $10^3$/µL | 8.13 (3.06) | 2 (0.04%) |
| Platelet count, $10^3$/µL | 209.7 (70.8) | 1 (0.02%) |
| APTT, sec | 27.1 (4.4) | 552 (11.99%) |

Data are mean (standard deviation) unless specified otherwise.
ALT, alanine aminotransferase; APTT, activated partial thromboplastin time; AST, aspartate aminotransferase; BP, blood pressure.

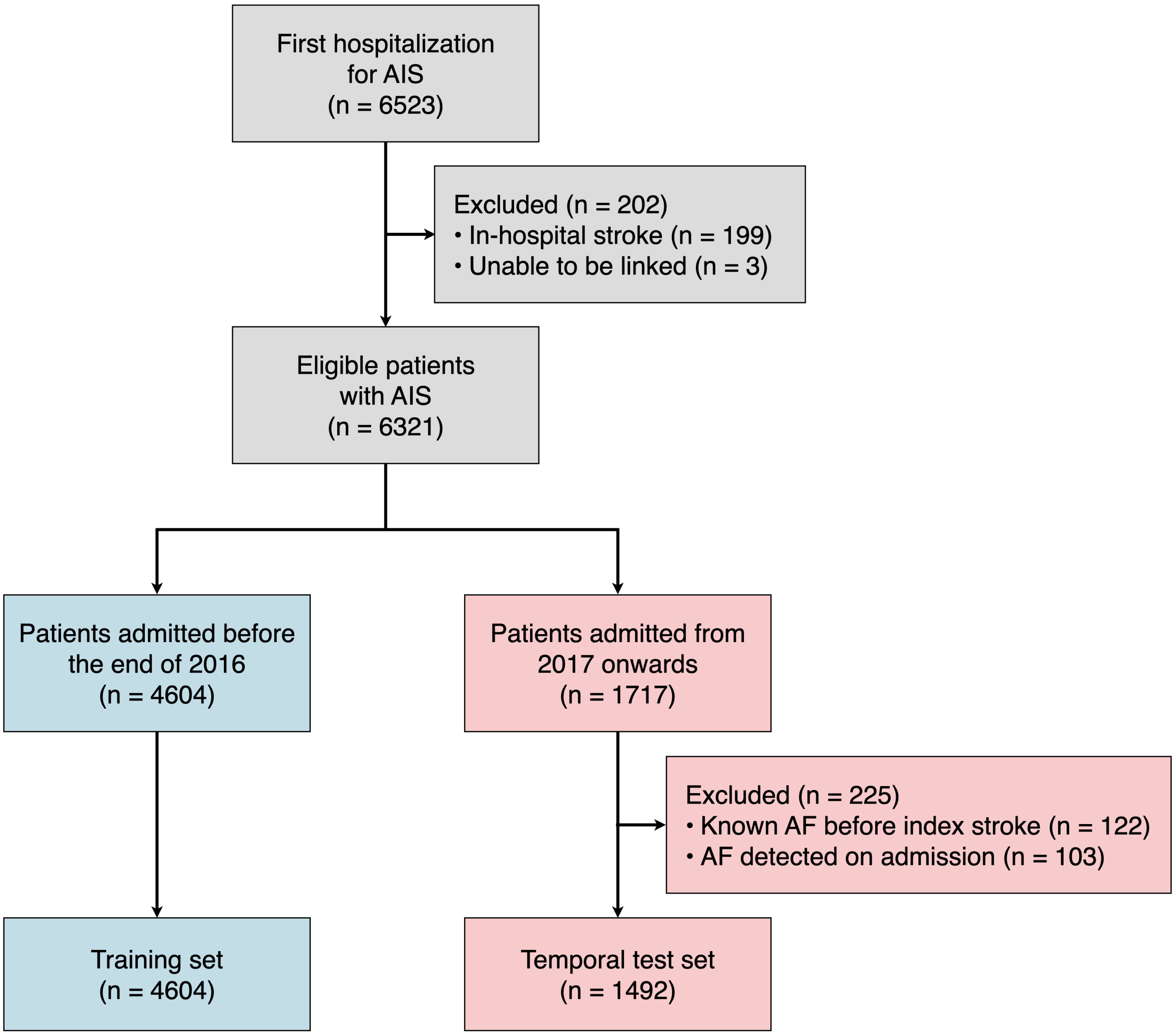


**Supplementary Figure 1.** Flowchart showing the derivation of the study cohorts. AF, atrial fibrillation; AIS, acute ischemic stroke.

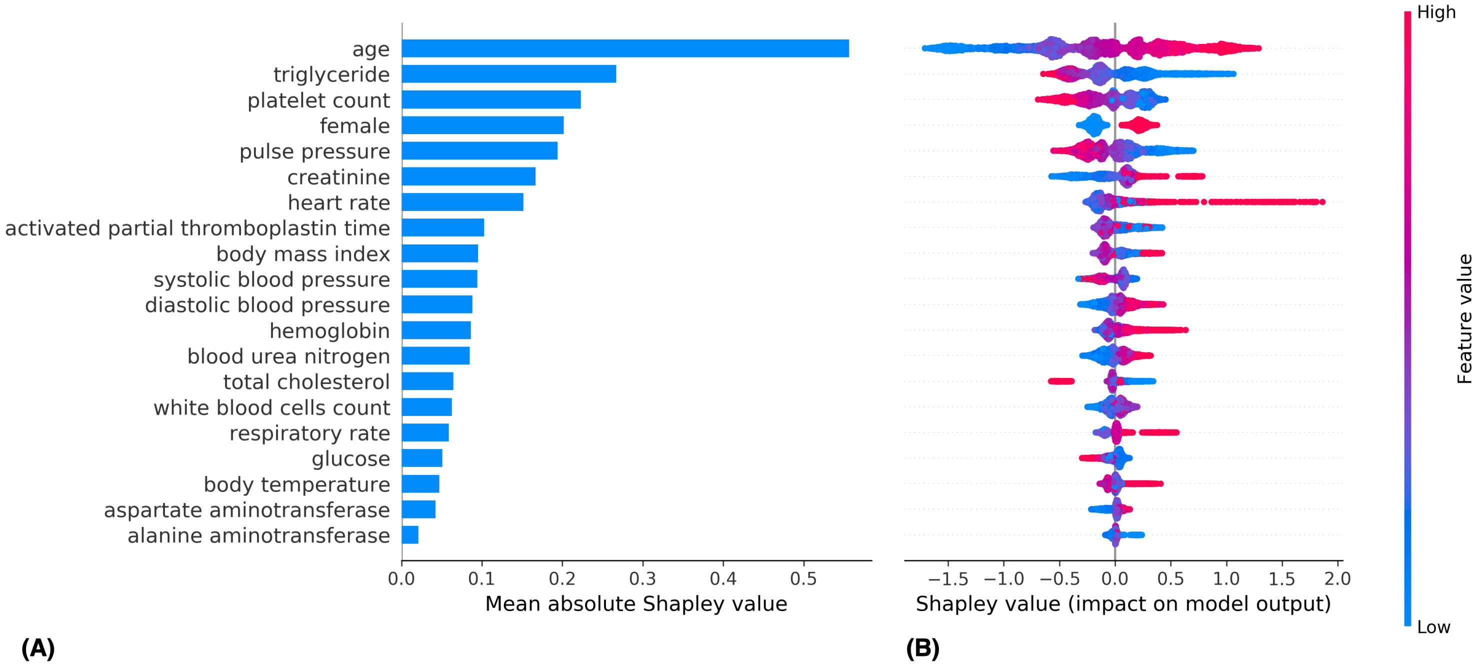


**Supplementary Figure 2.** The top 20 most important features identified by the model based on structured data (model A). The mean absolute Shapley values that indicate the average impact on model output are shown in a bar chart (A). The individual Shapley values for these features for each patient are depicted in a beeswarm plot (B), where a dot's position on the x-axis denotes each feature's contribution to the model prediction for that patient. The color of the dot specifies the relative value of the corresponding feature.

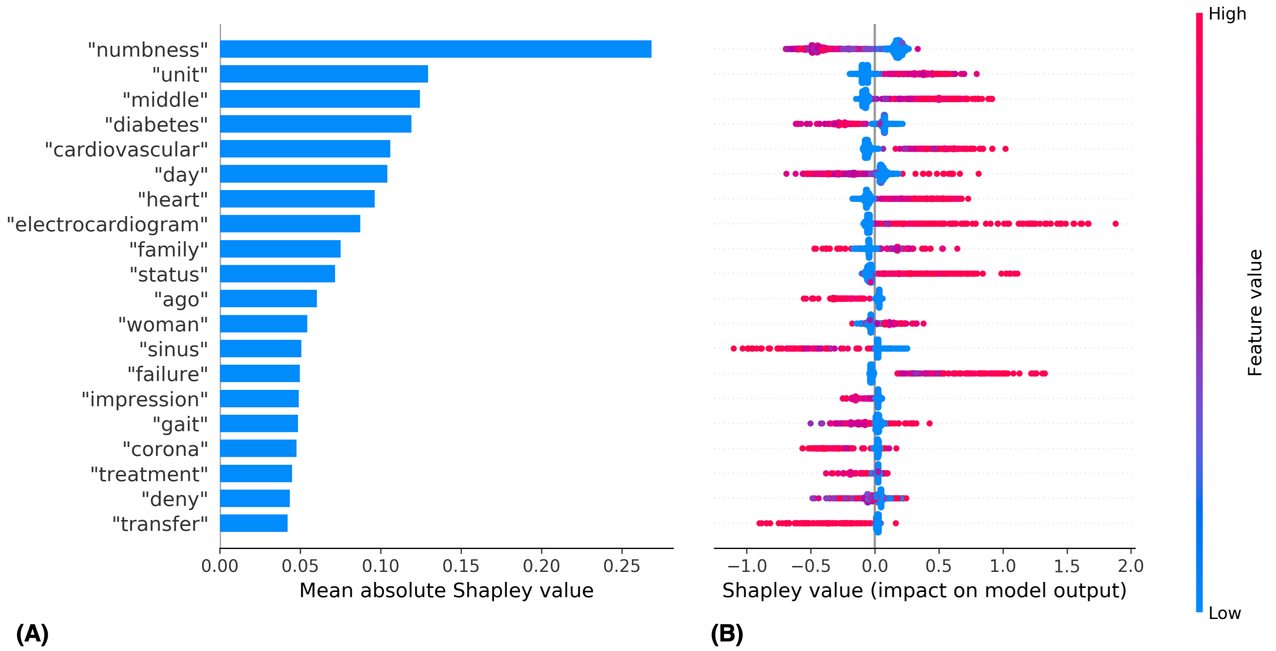


**Supplementary Figure 3.** The top 20 most important features identified by the model based on textual data vectorized using bag-of-words with term frequency with inverse document frequency weighting (model B). The mean absolute Shapley values that indicate the average impact on model output are shown in a bar chart (A). The individual Shapley values for these features for each patient are depicted in a beeswarm plot (B), where a dot's position on the x-axis denotes each feature's contribution to the model prediction for that patient. The color of the dot specifies the relative value of the corresponding feature.

## Supplementary References

1. Khurshid S, Keaney J, Ellinor PT, Lubitz SA. A Simple and Portable Algorithm for Identifying Atrial Fibrillation in the Electronic Medical Record. *Am J Cardiol.* (2016) 117:221–225. doi: 10.1016/j.amjcard.2015.10.031

2. Chapman WW, Bridewell W, Hanbury P, Cooper GF, Buchanan BG. A simple algorithm for identifying negated findings and diseases in discharge summaries. *J Biomed Inform.* (2001) 34:301–310. doi: 10.1006/jbin.2001.1029
